# Mechanisms and Intervention Strategies for Heat Stroke-Associated Myocardial Dysfunction: A Narrative Review

**DOI:** 10.5811/westjem.53045

**Published:** 2026-05-13

**Authors:** Yan Zhuang, Xiao-huan Zhuang, Xin-yuan Zhang, Da-Cheng Wang, Yan Yang

**Affiliations:** * Affiliated Hospital of Nanjing University of Chinese Medicine, Department of Critical Care Medicine, Nanjing, Jiangsu, China; † Jiangsu Province Hospital of Chinese Medicine, Department of Critical Care Medicine, Nanjing, Jiangsu, China

## Abstract

**Introduction:**

Heatstroke is a life-threatening condition defined by a core body temperature exceeding 40° C and central nervous system dysfunction. Its onset is potentiated by high heat and humidity, especially if superimposed upon high thermal loads due to exertion or to impaired ability to sweat as associated with the use of certain medications. The condition can trigger systemic inflammation and potentially fatal multi-organ failure. The heart is a primary organ affected; heatstroke-associated myocardial dysfunction may present as tachycardia, arrhythmias, heart failure, or ischemic injury.

**Methods:**

This narrative review was informed by a structured search of PubMed and Embase. The search focused on literature from the past 10 years, supplemented by earlier seminal studies where necessary. Key search terms included heatstroke, myocardial injury, dysfunction, biomarkers, cooling strategies, monitoring, and circulatory support. We prioritized human clinical studies, reviews, and consensus statements on acute management, along with preclinical studies.

**Results:**

Heatstroke-associated myocardial dysfunction has a multifactorial pathophysiology involving direct thermal cytotoxicity, systemic inflammation, endothelial injury, coagulopathy, mitochondrial dysfunction, and dysregulated cell death. Cardiac manifestations include myocardial injury, arrhythmia, and ventricular dysfunction. Early diagnosis requires an electrocardiogram, cardiac biomarkers, and echocardiography. Management is centered on rapid cooling, hemodynamic support, and close monitoring. Refractory cases may require invasive temperature control or mechanical circulatory support.

**Conclusion:**

Heatstroke-associated myocardial dysfunction is a clinically important and potentially reversible complication. Timely cooling, vigilant cardiovascular assessment, and supportive management remain central to care, while targeted therapies and refined risk-stratification strategies require further clinical investigation.

## INTRODUCTION

Global warming and related environmental changes have driven an increase in the number of extreme heat events, which currently account for an estimated 490,000 annual deaths worldwide. Modeling studies indicate a pronounced increase in heat-related fatalities, with projections suggesting a near twofold rise in global mortality between 2030–2050.^1^–^3^ Heatstroke, a life-threatening condition defined by a core body temperature > 40 °C (104 °F) accompanied by central nervous system dysfunction, is commonly classified into two subtypes based on etiology: classic heatstroke, triggered by passive heat exposure; and exertional heatstroke, associated with physical exertion.^4^

Cardiac dysfunction is a prevalent complication of heatstroke, affecting up to 65.2% of patients with multiorgan failure. Heatstroke-associated myocardial dysfunction (HS-MD) encompasses a broad spectrum of electrical and mechanical abnormalities, including sinus tachycardia, atrial and ventricular arrhythmias, and conduction disturbances.^5^,^6^ Elevations in cardiac troponins and natriuretic peptides are frequently observed and are associated with adverse outcomes. Emerging evidence suggests that endothelial-derived and extracellular vesicle–related biomarkers, such as von Willebrand factor, soluble thrombomodulin, and histone H3, may further refine risk stratification; however, heterogeneity in sampling windows, assay platforms, and outcome definitions limits direct comparability across studies.

Prompt recognition of myocardial involvement is, therefore, critical. Early, staged interventions—including rapid cooling, hemodynamic-guided resuscitation, and continuous electrocardiographic and echocardiographic monitoring—are essential. Timely escalation to advanced temperature control or temporary mechanical circulatory support may be life-saving; in selected cases, reversible myocardial depression has been reported.^7^ In this narrative review we aimed to synthesize the clinical manifestations and pathophysiological mechanisms of HS-MD, critically appraise current and emerging biomarkers, and propose a pragmatic, time-windowed management framework, while outlining priorities for future research.

## METHODS

### Literature Search Strategy

This is a narrative review supported by a structured literature search. We searched PubMed and Embase, primarily focusing on studies published within the past 10 years, while also including selected earlier landmark studies where necessary to provide essential background. Our search strategy combined terms related to heatstroke and cardiac involvement, including (“heatstroke” OR “heat stroke” OR “exertional heat stroke” OR “classic heat stroke”) AND (myocard* OR cardiac OR “myocardial injury” OR “myocardial dysfunction” OR arrhythmia OR troponin OR echocardiograph*) AND (cooling OR resuscitation OR monitoring OR vasopressor* OR “mechanical circulatory support”). We also screened reference lists of relevant reviews and consensus statements to identify additional pertinent studies.

We prioritized human clinical studies, narrative reviews, and guidelines or consensus statements relevant to the pathophysiology and acute management of HS-MD. Selected preclinical studies were included when they provided mechanistic insights with potential clinical relevance. We excluded non English-language articles without accessible full text and studies not related to cardiac involvement in heatstroke.

### Clinical Characteristics of Heatstroke-associated Myocardial Dysfunction

#### Arrhythmias

Within 24 hours of heatstroke onset, patients may develop a range of electrocardiographic (ECG) abnormalities, including sinus tachycardia, ventricular tachycardia, QT-interval prolongation, diffuse nonspecific ST–T changes, and ST–T alterations suggestive of myocardial ischemia.^8^ The incidence of atrial arrhythmias has been reported to reach up to 24%, while severe cases may present with ventricular tachycardia or even cardiac arrest.^9^,^10^

Atrioventricular conduction disturbances associated with heatstroke include PR-interval prolongation, intraventricular conduction delays, and left or right bundle branch block.^11^ QT- interval prolongation is a particularly common finding and may be partially attributable to electrolyte disturbances induced by heatstroke.^12^ Beyond the acute phase, heatstroke has also been associated with increased long-term cardiovascular risk.^9^,^13^ A 14-year follow-up study of discharged patients demonstrated a 3.9-fold increase in major adverse cardiovascular events and a 15-fold increase in the incidence of atrial fibrillation.^14^

#### Myocardial Injury

At the molecular level, heatstroke can exert persistent effects on cellular function and epigenetic regulation, which may remain subclinical until unmasked by subsequent stressors or aging-related processes.^15^,^16^ Clinically, myocardial injury related to heatstroke poses a diagnostic challenge, as it can closely mimic acute coronary syndromes. Typical findings include territory-like ST-segment deviations and marked T-wave inversions on ECG.^9^

Elevations in cardiac troponin levels are common and carry important prognostic implications, with severe elevations associated with increased one-year mortality.^17^ Importantly, this pattern of myocardial injury is thought to reflect supply–demand mismatch and microvascular dysfunction rather than acute coronary thrombosis. Accordingly, a comprehensive diagnostic approach integrating clinical presentation, echocardiographic assessment, and biomarker kinetics is essential to distinguish HS-MD from type 1 (atherothrombotic) myocardial infarction.

##### Heart Dysfunction

Heat exposure leads to reductions in circulating blood volume and systemic vascular resistance, prompting compensatory increases in cardiac output to maintain blood pressure, tissue perfusion, and thermal homeostasis. Experimental and clinical studies have shown that for every 0.3 °C increase in core body temperature, myocardial contractility and cardiac output rise, while perfusion to certain organs decreases and cutaneous vasodilation intensifies to facilitate heat dissipation.^18^,^19^

Older adults and patients with pre-existing cardiac disease often exhibit impaired cutaneous vasodilation and diminished cardiac reserve, rendering them particularly vulnerable to circulatory failure during heat stress. Extreme heat is associated with increased mortality across all age groups, with individuals with chronic diseases—especially cardiovascular disease—at disproportionately higher risk.^20^ A multinational analysis across 27 countries reported that heat-related heart failure accounted for approximately 0.26% of cardiovascular deaths.^21^ In addition, epidemiological data indicate that extreme heat exposure is associated with a 12% increase in heart failure–related mortality.^22^

### Pathophysiology of Heatstroke-Associated Myocardial Dysfunction

Heatstroke-associated myocardial dysfunction results from the combined effects of direct thermal injury and systemic physiological derangements induced by extreme hyperthermia. Epidemiological studies consistently associate heat exposure with increased cardiovascular morbidity and mortality; however, myocardial impairment in heatstroke differs fundamentally from primary ischemic heart disease and is often functional, dynamic, and potentially reversible.^23^–^25^

#### Direct Thermal Injury to Cardiomyocytes

At the cellular level, extreme hyperthermia exerts direct cytotoxic effects on cardiomyocytes. Elevated core temperatures disrupt protein conformation, impair calcium homeostasis, and alter membrane integrity, leading to reduced myocardial contractility and electrical instability.^1^,^20^ These temperature-dependent changes provide a mechanistic explanation for the frequent occurrence of arrhythmias, transient systolic dysfunction, and repolarization abnormalities observed during acute heatstroke.^26^,^27^

#### Systemic Inflammation and Endothelial Dysfunction

Beyond direct thermal effects, heatstroke provokes a systemic inflammatory response that resembles sepsis-like physiology. Heat-induced cellular injury triggers widespread inflammatory activation, accompanied by endothelial dysfunction and increased vascular permeability. These processes promote microvascular dysregulation and impair effective tissue perfusion.^28^,^29^ Within the myocardium, inflammatory mediators and endothelial injury compromise oxygen delivery and myocardial energetics, contributing to myocardial depression even in the absence of obstructive coronary artery disease.^30^–^32^

#### Microcirculatory and Metabolic Disturbances

Microcirculatory impairment further exacerbates myocardial dysfunction in heatstroke.^33^ Hypovolemia, peripheral vasodilation, and endothelial injury reduce effective circulating volume and limit coronary microvascular perfusion.^34^ In parallel, metabolic disturbances—particularly mitochondrial dysfunction and impaired adenosophine triphosphate generation—restrict the heart’s ability to meet increased metabolic demands during heat stress. Together, these alterations predispose susceptible patients to acute heart failure, shock, or circulatory collapse.^35^

#### Reversibility and Clinical Implications

**Figure 1:**
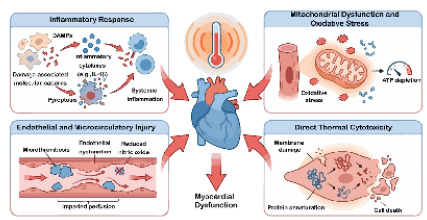
Pathophysiology of heatstroke-associated myocardial dysfunction. Extreme hyperthermia leads to myocardial dysfunction through four inter-related pathophysiological mechanisms.1) inflammatory response: heat-induced cellular injury triggers release of damage-associated molecular patterns and pro-inflammatory cytokines, resulting in systemic inflammation and cardiomyocyte injury. 2) Mitochondrial dysfunction and oxidative stress: excessive heat and metabolic stress impair mitochondrial function, promote oxidative stress, and reduce energy production, contributing to myocardial depression and electrical instability. 3) Endothelial and microcirculatory injury: endothelial dysfunction, reduced nitric oxide bioavailability, and microthrombosis impair myocardial perfusion and exacerbate ischemia-like injury. 4) Direct thermal cytotoxicity: extreme temperatures cause protein denaturation, membrane damage, and cardiomyocyte death. These mechanisms converge to produce clinically relevant myocardial dysfunction, which is often functional and potentially reversible with timely intervention.

Importantly, myocardial dysfunction in heatstroke is frequently reversible with timely intervention. Rapid temperature reduction, restoration of circulating volume and perfusion, and attenuation of systemic inflammation may lead to recovery of cardiac function over hours to days. This predominantly functional and inflammatory pattern of myocardial injury highlights the importance of early recognition and aggressive supportive management, rather than therapeutic strategies aimed at irreversible structural myocardial damage.^6^,^12^,^36^ A schematic overview of the principal mechanistic pathways involved in HS-MD is presented in Figure 1.

### Management of Heatstroke-Associated Myocardial Dysfunction

#### Early Recognition and Risk Stratification

Early recognition of myocardial involvement is essential in patients with heatstroke, as timely identification informs monitoring intensity, disposition, and subsequent management. Clinical suspicion should be heightened in the presence of arrhythmias, hemodynamic instability, or signs of cardiac dysfunction.^45^ Electrocardiography remains a first-line tool for detecting conduction abnormalities and malignant arrhythmias and should be performed early in the course of evaluation.^11^,^46^,^47^

Cardiac biomarkers play an important adjunctive role in risk stratification. Elevations in cardiac troponins are common in heatstroke and may reflect myocardial injury related to supply–demand mismatch and microvascular dysfunction rather than acute coronary thrombosis.^37^,^48^ When interpreted dynamically, troponin trends can provide prognostic information and help differentiate transient functional myocardial injury from evolving ischemic events. Natriuretic peptides, including B-type natriuretic peptide, may further assist in identifying patients with myocardial stress or overt cardiac dysfunction.^49^

Markers of endothelial injury and inflammation have also been explored as potential tools for early risk assessment. Circulating endothelial cells, adhesion molecules, thrombomodulin, and von Willebrand factor antigens may reflect microvascular injury and endothelial dysfunction in severe heatstroke. Although these biomarkers are not routinely available in all clinical settings, their presence highlights the systemic vascular involvement underlying heatstroke-associated myocardial dysfunction. When available, bedside echocardiography provides complementary information by assessing ventricular function, preload status, and potential reversible myocardial depression. Integration of clinical findings, ECG changes, biomarker trends, and echocardiographic assessment allow a more comprehensive and clinically meaningful stratification of cardiovascular risk in patients with heatstroke.

#### Rapid Temperature Control: Principles and Comparative Strategies

Rapid reduction of core body temperature remains the cornerstone of heatstroke management and is particularly critical in patients with suspected HS-MD.^50^ The magnitude and duration of hyperthermia are closely associated with myocardial injury, arrhythmias, and hemodynamic compromise; therefore, cooling should be initiated as early as possible and continued until target temperature is achieved.

#### Goals and Timing of Cooling

The primary objective of therapeutic cooling is the rapid reduction of core temperature to below 39° C, followed by maintenance to prevent rebound hyperthermia.^51^ A cooling rate exceeding 0.15 °C/minute is critical for survival and is associated with significantly reduced fatality and complication rates.^52^ Clinical outcomes exhibit a clear time-dependent relationship with cooling speed; delays in temperature reduction are linked to more severe neurological and cardiovascular injury. In patients with pre-existing myocardial dysfunction, inadequate or delayed cooling can exacerbate myocardial depression and trigger malignant arrhythmias.

#### External Cooling Methods

External cooling techniques are considered first-line therapy in most patients with heatstroke. Immediate cooling measures were implemented, which included giving cold intravenous fluids, placing ice packs, using lukewarm water and fans, applying wet towels, and using full-body medical cooling suits such as CarbonCool.^53^,^54^ Ice-water immersion achieves the most rapid cooling rates and is particularly effective in exertional heatstroke.^55^,^56^

However, its use may be limited in patients with altered mental status, advanced age, or cardiovascular instability. Evaporative and convective cooling methods, including mist-and-fan techniques and surface cooling devices, are more commonly employed in emergency and intensive care settings. Although these methods may achieve slower cooling rates, they allow improved access for airway management, cardiovascular monitoring, and hemodynamic support, which is especially relevant in patients with myocardial involvement.^18^,^57^

#### Intravascular and Invasive Cooling

Intravascular or other invasive cooling strategies may be considered when external cooling measures fail to achieve adequate temperature control or are contraindicated. The available evidence supporting these approaches in heatstroke is limited and largely anecdotal. Reported cases suggest that invasive cooling may facilitate effective temperature reduction in selected patients with severe or refractory hyperthermia.^58^ At present, these techniques should be regarded as rescue strategies rather than routine therapy, and their use should be individualized based on the patient’s condition, resource availability, and procedural risk.^59^

#### Hemodynamic Support and Volume Management

Hemodynamic management should be tailored to the presence and severity of myocardial dysfunction. Initial fluid resuscitation is often required to address hypovolemia; however, excessive fluid administration may exacerbate myocardial stress and pulmonary congestion in patients with impaired cardiac function.^12^ Dynamic assessment of volume responsiveness, integration of bedside echocardiography, and close monitoring of perfusion parameters are recommended. In patients with persistent hypotension despite appropriate volume resuscitation, vasopressor support may be required, with careful titration to maintain end-organ perfusion while avoiding excessive afterload.^6^,^18^

#### Cardiovascular Monitoring and Support

Continuous cardiovascular monitoring is recommended in patients with suspected HS-MD. Continuous ECG monitoring enables early detection and prompt management of arrhythmias, which may occur during both the hyperthermic phase and the cooling process. Serial assessment of cardiac biomarkers may provide insight into the progression or resolution of myocardial injury. Echocardiography, when available, should be used to monitor ventricular function and guide ongoing hemodynamic management. Supportive care should focus on optimizing oxygen delivery, correcting electrolyte disturbances, and managing arrhythmias in accordance with standard critical care principles.^9^

#### Advanced and Rescue Therapies

In rare cases complicated by refractory shock or cardiac arrest, escalation to advanced supportive therapies may be considered. Mechanical circulatory support, such as venoarterial extracorporeal membrane oxygenation, has been reported in isolated cases of severe heatstroke with cardiovascular collapse.^60^,^61^ However, the available evidence remains limited, and patient selection should be individualized. Decisions regarding escalation to advanced support should consider the reversibility of organ dysfunction, neurological status, and overall prognosis.

#### Pharmacologic and Experimental Approaches

Several pharmacologic and experimental strategies, including antioxidant therapies and metabolic modulators such as L-carnitine, have been explored primarily in preclinical or experimental settings.^62^,^63^ Although these approaches offer mechanistic insights into potential myocardial protection during hyperthermia, their clinical efficacy and safety in HS-MD have not been established. At present, such strategies should be considered investigational and should not replace established temperature-directed and supportive management.^3^

## LIMITATIONS

This review has several limitations that should be acknowledged. First, as a narrative rather than a systematic review, the study is subject to selection and publication bias, and the included literature may not comprehensively represent all available evidence. Second, although a structured literature search was performed, we did not conduct formal study quality assessment or quantitative synthesis, which limits direct comparison across studies. Third, heterogeneity in study designs, patient populations, outcome definitions, and biomarker assays precludes firm conclusions regarding causality or the relative importance of specific mechanisms. Finally, much of the mechanistic evidence is derived from preclinical or observational studies, and its direct applicability to emergency and critical care practice requires further validation. These limitations underscore the need for well-designed prospective studies and translational research in this evolving field.

## CONCLUSION

Heatstroke-associated myocardial dysfunction is a frequent and clinically significant complication of severe hyperthermia that is typically functional, inflammatory, and potentially reversible rather than ischemic in nature.^57^ Early recognition, prompt temperature control, individualized hemodynamic support, and continuous cardiovascular monitoring remain the cornerstones of management in emergency and critical care settings. Improved awareness and further clinical studies are needed to refine risk stratification and optimize supportive strategies for this vulnerable patient population.

## Figures and Tables

**Table 1:** Biomarkers for heatstroke-associated myocardial dysfunction, analytical characteristics, typical heatstroke patterns, and clinical utility.

Biomarker	Pathway / what it reflects	Preferred sampling and units	Typical HS pattern	Prognostic / clinical use	Pitfalls, availability and turnaround	Key refs
hs-cTnI/T	Myocyte injury (thermal stress, microvascular dysfunction, supply–demand mismatch)	0 h, 3–6 h, 12–24 h; assay-specific 99th percentile and delta	Transient elevation after hyperthermia; may decline with effective cooling	Severity signal; predicts short- and long-term mortality; integrate with ECG/echo to avoid misclassification as type-1 MI	Widely available in ED; rapid turnaround (≈1 h). Non-ACS elevations common in HS; influenced by exertion and renal dysfunction	37
BNP / NT-proBNP	Ventricular wall stress and dysfunction	0 h and 3–24 h; pg/mL	Frequently elevated; tracks LV dysfunction and volume stress	Complements echocardiography for triage, disposition, and prognosis	ED/ICU-available; turnaround ≈1–2 h. Affected by age, renal function, and atrial arrhythmias	38
Serum myoglobin (sMb) / LDH	Myocyte necrosis and systemic ischemic injury	sMb ≥1000 ng/mL; LDH elevated	Markedly elevated in severe HS, often with rhabdomyolysis	Better predictor of AKI and 90-day mortality than CK in EHS	Widely available; turnaround ≈1–2 h. Limited cardiac specificity; influenced by skeletal muscle injury	39
Syndecan-1	Endothelial glycocalyx shedding	0–24 h; ng/mL	Elevated in moderate–severe HS	Correlates with organ dysfunction; surrogate of vascular injury	Research-oriented; limited ED availability; delayed turnaround; not myocardium-specific	40
Platelet count, PT/INR, fibrinogen, D-dimer	HS-induced coagulopathy / DIC overlap	Baseline and serial (6–12 h)	Thrombocytopenia, low fibrinogen, elevated D-dimer	Stage coagulopathy; guide monitoring and escalation	Widely available; rapid turnaround. Affected by transfusion, hypothermia, liver dysfunction	39
IL-6 / HMGB1	Systemic inflammation and DAMP release	Early (0–6 h) and trend	Peaks around hyperthermia or early cooling	Global severity adjunct; may signal escalation	Limited routine availability; delayed turnaround; non-specific	41
Circulating endothelial cells	Endothelial injury	0–24 h; specialized platforms	Elevated with severe endothelial damage	Research use; potential risk stratifier	Specialized assays only; pre-analytical variability	42,43
EV-histone H3	NET-related cellular injury	0–24 h; exploratory assays	Elevated in severe HS	Early severity signal (exploratory)	Research-only; assay standardization lacking	44

Biomarkers listed in this table vary substantially in clinical availability and turnaround time. Cardiac troponins, natriuretic peptides, and routine coagulation parameters are widely available in most emergency departments and can typically be obtained within 1–2 hours, making them the most actionable tools for acute risk stratification. In contrast, markers of endothelial injury and inflammation (e.g., syndecan-1, IL-6, HMGB1, circulating endothelial cells, EV–histone H3) are primarily research-based, often require specialized assays, and are not routinely available for real-time emergency decision-making. Reported biomarker patterns should be interpreted in the clinical context of hyperthermia, exertion, renal function, and concomitant organ dysfunction. Elevations do not necessarily indicate primary ischemic heart disease and should be integrated with electrocardiography and echocardiography to guide management.

*h*, hours*; HS*, heatstroke; *HS-MD*, heatstroke-associated myocardial dysfunction; *hs-cTnI/T*, high-sensitivity cardiac troponin I/T; *BNP*, B-type natriuretic peptide; *LDH*, lactate dehydrogenase; *AKI*, acute kidney injury; *DIC*, disseminated intravascular coagulation; *DAMPs*, danger-associated molecular patterns; *EV*, extracellular vesicle.

## References

[1] Desai Y, Khraishah H, Alahmad B (2023). Heat and the heart. Yale J Biol Med.

[2] Romanello M, Di Napoli C, Drummond P (2022). The 2022 report of the *Lancet* Countdown on Health and Climate Change: health at the mercy of fossil fuels. Lancet.

[3] Kew MC, Tucker RB, Bersohn I (1969). The heart in heatstroke. Am Heart J.

[4] Bouchama A, Abuyassin B, Lehe C (2022). Classic and exertional heatstroke. Nat Rev Dis Primers.

[5] Garcia CK, Renteria LI, Leite-Santos G (2022). Exertional heat stroke: pathophysiology and risk factors. BMJ Med.

[6] Peters A, Schneider A (2021). Cardiovascular risks of climate change. Nat Rev Cardiol.

[7] Marchand M, Gin K (2022). The cardiovascular system in heat stroke. CJC Open.

[8] Shimada M, Tsai B, Marshall JP (2015). A case of heatstroke complicated by persistent ventricular tachycardia. J Emerg Med.

[9] Paul A, Alex R, Jacob JR (2019). Effects of heat stroke on surface ECG: a study on clinical outcomes. Heart Asia.

[10] Petropoulos ZE, Keogh SA, Jarquín E (2023). Heat stress and heat strain among outdoor workers in El Salvador and Nicaragua. J Expo Sci Environ Epidemiol.

[11] Mimish L (2012). Electrocardiographic findings in heat stroke and exhaustion: a study on Makkah pilgrims. J Saudi Heart Assoc.

[12] Bathini T, Thongprayoon C, Petnak T (2020). Circulatory failure among hospitalizations for heatstroke in the United States. Medicines (Basel).

[13] Singh N, Areal AT, Breitner S (2024). Heat and cardiovascular mortality: an epidemiological perspective. Circ Res.

[14] Wang J-C, Chien W-C, Chu P (2019). The association between heat stroke and subsequent cardiovascular diseases. PLoS One.

[15] Weyrich A, Lenz D, Jeschek M (2016). Paternal intergenerational epigenetic response to heat exposure in male wild guinea pigs. Mol Ecol.

[16] Velichko AK, Petrova NV, Razin SV (2015). Mechanism of heat stress-induced cellular senescence elucidates the exclusive vulnerability of early S-phase cells to mild genotoxic stress. Nucleic Acids Res.

[17] Dervišević E, Hasić S, Katica M (2023). Heat-related biomarkers: focus on the correlation of troponin I and 70 kDa heat shock protein. Heliyon.

[18] Crandall CG, González-Alonso J (2010). Cardiovascular function in the heat-stressed human. Acta Physiol (Oxf).

[19] Crandall CG, Wilson TE, Marving J (2008). Effects of passive heating on central blood volume and ventricular dimensions in humans. J Physiol.

[20] Singh N, Areal AT, Breitner S (2024). Heat and cardiovascular mortality: an epidemiological perspective. Circ Res.

[21] Alahmad B, Khraishah H, Royé D (2023). Associations between extreme temperatures and cardiovascular cause-specific mortality: results from 27 countries. Circulation.

[22] Khraishah H, Alahmad B, Ostergard RL (2022). Climate change and cardiovascular disease: implications for global health. Nat Rev Cardiol.

[23] Bauman J, Spano S, Storkan M (2024). Heat-related illnesses. Emerg Med Clin North Am.

[24] Cramer MN, Gagnon D, Laitano O (2022). Human temperature regulation under heat stress in health, disease, and injury. Physiol Rev.

[25] Wang Z, Zhu J, Zhang D (2024). The significant mechanism and treatments of cell death in heatstroke. Apoptosis.

[26] De Vita A, Belmusto A, Di Perna F (2024). The impact of climate change and extreme weather conditions on cardiovascular health and acute cardiovascular diseases. J Clin Med.

[27] Ferrer R, Iba T (2025). Molecular mechanisms of heatstroke: pathophysiology and cell death pathways. Juntendo Med J.

[28] Bouchama A, Abuyassin B, Lehe C (2022). Classic and exertional heatstroke. Nat Rev Dis Primers.

[29] Chen J, Ding C, Cao J (2023). Heat stress combined with lipopolysaccharide induces pulmonary microvascular endothelial cell glycocalyx inflammatory damage in vitro. Immun Inflamm Dis.

[30] Yamaga S, Aziz M, Murao A (2024). DAMPs and radiation injury. Front Immunol.

[31] Bouchama A, Roberts G, Al Mohanna F (2005). Inflammatory, hemostatic, and clinical changes in a baboon experimental model for heatstroke. J Appl Physiol (1985).

[32] Tsai H-Y, Hsu Y-J, Lu C-Y (2021). Pharmacological activation of aldehyde dehydrogenase 2 protects against heatstroke-induced acute lung injury by modulating oxidative stress and endothelial dysfunction. Front Immunol.

[33] Iba T, Maier CL, Levi M (2024). Thromboinflammation and microcirculation damage in heatstroke. Minerva Med.

[34] Kravitz MS, Lee JH, Shapiro NI (2024). Cardiac arrest and microcirculatory dysfunction: a narrative review. Curr Opin Crit Care.

[35] Zhou B, Tian R (2018). Mitochondrial dysfunction in pathophysiology of heart failure. J Clin Invest.

[36] Laitano O, Garcia CK, Mattingly AJ (2020). Delayed metabolic dysfunction in myocardium following exertional heat stroke in mice. J Physiol.

[37] Dervišević E, Hasić S, Katica M (2023). Heat-related biomarkers: focus on the correlation of troponin I and 70 kDa heat shock protein. Heliyon.

[38] Schlader ZJ, Davis MS, Bouchama A (2022). Biomarkers of heatstroke-induced organ injury and repair. Exp Physiol.

[39] Wu M, Wang C, Zhong L (2022). Serum myoglobin as predictor of acute kidney injury and 90-day mortality in patients with rhabdomyolysis after exertional heatstroke: an over 10-year intensive care survey. Int J Hyperthermia.

[40] Kobayashi K, Mimuro S, Sato T (2018). Dexmedetomidine preserves the endothelial glycocalyx and improves survival in a rat heatstroke model. J Anesth.

[41] Palasz J, Farooqi W, Musharraf MB (2025). Diagnostic biomarkers for heat stroke and heat exhaustion: a scoping review. Disaster Med Public Health Prep.

[42] Truong SK, Katoh T, Mimuro S (2021). Inhalation of 2% hydrogen improves survival rate and attenuates shedding of vascular endothelial glycocalyx in rats with heat stroke. Shock.

[43] Iba T, Maier CL, Levi M (2024). Thromboinflammation and microcirculation damage in heatstroke. Minerva Med.

[44] Murray KO, Clanton TL, Horowitz M (2022). Epigenetic responses to heat: from adaptation to maladaptation. Exp Physiol.

[45] Roberts WO, Armstrong LE, Sawka MN (2023). ACSM expert consensus statement on exertional heat illness: recognition, management, and return to activity. Curr Sports Med Rep.

[46] Zhang Z, Wu X, Zou Z (2024). Heat stroke: pathogenesis, diagnosis, and current treatment. Ageing Res Rev.

[47] Bao C-H, Feng Q, Zhang C (2024). Heat stroke with significantly elevated troponin and dynamic ECG changes: myocardial infarction or myocardial injury?. Am J Med Sci.

[48] Palasz J, Farooqi W, Musharraf MB (2025). Diagnostic biomarkers for heat stroke and heat exhaustion: a scoping review. Disaster Med Public Health Prep.

[49] Feng L, Yin J-Y, Liu Y-H (2024). N-terminal pro-brain natriuretic peptide - a significant biomarker of disease development and adverse prognosis in patients with exertional heat stroke. Mil Med Res.

[50] Chen L, Xu S, Yang X (2023). Association between cooling temperature and outcomes of patients with heat stroke. Intern Emerg Med.

[51] DeGroot DW, Ruby B, Koo A (2025). Far from home: heat-illness prevention and treatment in austere environments. Wilderness Environ Med.

[52] Cong S, Zheng G, Liang X (2025). Pre-hospital cooling in community-acquired heat stroke (CAHS): evidence, challenges, and strategies. Eur J Med Res.

[53] Rublee C, Dresser C, Giudice C (2021). Evidence-based heatstroke management in the emergency department. West J Emerg Med.

[54] Kido N, Tagami T, Otake K (2024). Exploring the potential of CarbonCool® in rapid prehospital cooling for severe heat stroke. Prehosp Emerg Care.

[55] Barletta JF, Palmieri TL, Toomey SA (2024). Management of heat-related illness and injury in the ICU: a concise definitive review. Crit Care Med.

[56] Hosokawa Y, Racinais S, Akama T (2021). Prehospital management of exertional heat stroke at sports competitions: International Olympic Committee adverse weather impact expert working group for the Olympic Games Tokyo 2020. Br J Sports Med.

[57] Liu J, Varghese BM, Hansen A (2022). Heat exposure and cardiovascular health outcomes: a systematic review and meta-analysis. Lancet Planet Health.

[58] Bursey MM, Galer M, Oh RC (2019). Successful management of severe exertional heat stroke with endovascular cooling after failure of standard cooling measures. J Emerg Med.

[59] Hamaya H, Hifumi T, Kawakita K (2015). Successful management of heat stroke associated with multiple-organ dysfunction by active intravascular cooling. Am J Emerg Med.

[60] Chizhikova IO, Shigeev SV, Gornostaev DV (2025). A rare case of rhabdomyolysis due to heatstroke in an athlete. Sud Med Ekspert.

[61] Allen SB, Cross KP (2014). Out of the frying pan, into the fire: a case of heat shock and its fatal complications. Pediatr Emerg Care.

[62] Goto H, Nakashima H, Mori K (2024). L-carnitine pretreatment ameliorates heat stress-induced acute kidney injury by restoring mitochondrial function of tubular cells. Am J Physiol Renal Physiol.

[63] Wang X, Liu Y, Zhang C (2021). Protective effect of L-carnitine on myocardial injury in rats with heatstroke. Acta Cir Bras.

